# Brain Glucose Hypometabolism and Brain Iron Accumulation as Therapeutic Targets for Alzheimer’s Disease and Other CNS Disorders

**DOI:** 10.3390/ph18020271

**Published:** 2025-02-19

**Authors:** Indira Y. Rao, Leah R. Hanson, William H. Frey

**Affiliations:** 1 HealthPartners Center for Memory and Aging, Saint Paul, MN 55130, USA; rao00111@umn.edu (I.Y.R.); leah.r.hanson@healthpartners.com (L.R.H.); 2 HealthPartners Institute, Bloomington, MN 55425, USA

**Keywords:** brain glucose hypometabolism, brain iron accumulation, Alzheimer’s disease, intranasal insulin, intranasal deferoxamine, intranasal treatment, neuroinflammation, brain cell energy, nose-to-brain delivery

## Abstract

Two common mechanisms contributing to multiple neurological disorders, including Alzheimer’s disease, are brain glucose hypometabolism (BGHM) and brain iron accumulation (BIA). Currently, BGHM and BIA are both widely acknowledged as biomarkers that aid in diagnosing CNS disorders, distinguishing between disorders with similar symptoms, and tracking disease progression. Therapeutics targeting BGHM and BIA in Alzheimer’s disease can be beneficial in treating neurocognitive symptoms. This review addresses the evidence for the therapeutic potential of targeting BGHM and BIA in multiple CNS disorders. Intranasal insulin, which is anti-inflammatory and increases brain cell energy, and intranasal deferoxamine, which reduces oxidative damage and inflammation, represent promising treatments targeting these mechanisms. Both BGHM and BIA are promising therapeutic targets for AD and other CNS disorders.

## 1. Introduction

Alzheimer’s disease (AD) is a neurodegenerative disorder characterized by progressive memory loss, impaired cognition, confusion, disorientation, and other symptoms associated with dementia. It is the most common neurodegenerative disorder and can severely impair the quality of patients’ lives. A 2024 report from the Alzheimer’s Association reported that 6.9 million Americans age 65 and older have AD, showing how prevalent the disorder is in older adults [1]. Caregivers of those with AD provided an estimated 18.4 billion hours of unpaid assistance in 2023, demonstrating the large financial burden and time-intensive nature of caregiving [1]. More recent research efforts have been focused on developing disease-modifying therapies that are specific to treating AD [2]. However, many treatments that have the potential to ameliorate symptoms of AD and other CNS disorders involving reduced brain cell energy and increased neuroinflammation have not received sufficient attention.

One of the primary reasons why there are few, if any, highly effective treatments being prescribed for patients with AD, is that the etiology of the disease is not completely understood. For many decades, the amyloid hypothesis has prevailed, which originally proposed that extracellular aggregates of amyloid beta initiate cellular pathways that ultimately lead to cell death [3]. However, several clinical trials of drugs that reduced amyloid beta plaques were not effective in treating AD. There are, however, two recent immunotherapy treatments, donanemab and lecanemab, that have been approved for AD although their safety and effectiveness are not entirely clear [4]. Currently, another prevalent hypothesis for what causes AD is the abnormal accumulation of hyperphosphorylated tau (p-tau). The neurofibrillary tangles that form with accumulation of p-tau have shown a stronger correlation with memory loss and disease progression than amyloid beta plaques and disease-modifying therapies for this hypothesis are being developed [5].

**Figure 1:**
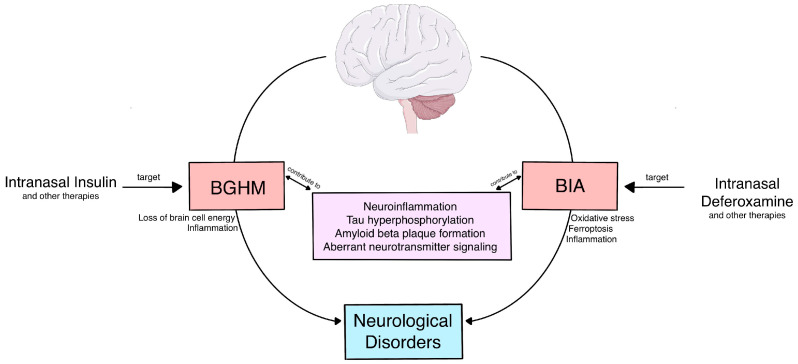
The relationship between brain glucose hypometabolism (BGHM), brain iron accumulation (BIA), and many other mechanisms involved in Alzheimer’s disease pathology as well as other neurological disorders, is outlined. Drugs targeting BGHM and BIA, such as intranasally administered insulin and deferoxamine, respectively, are promising therapies for Alzheimer’s disease and other neurological disorders as shown. (This schematic drawing was created using art elements from Servier Medical Art Commons Attribution 3.0 Unported License. Servier Medical Art by Servier is licensed under a Creative Commons Attribution 3.0 Unported License).

Although it is important to better understand what causes AD, there are also reasons to consider therapeutics that target common mechanisms across neurological disorders. Two common mechanisms contributing to multiple neurological disorders are brain glucose hypometabolism (BGHM) and brain iron accumulation (BIA) as described below (Figure 1). Currently, BGHM and BIA are both widely acknowledged as biomarkers that aid in diagnosing CNS disorders, distinguishing between those with similar symptoms, and tracking disease progression [6]. This narrative review expands on our previous paper [6] which reviewed the brain regions exhibiting BGHM and/or BIA in AD and Parkinson’s Disease (PD). We address the evidence for the therapeutic potential of targeting BGHM and BIA, the many neurodegenerative disease mechanisms in which these biomarkers are involved, and treatments that target each.

### 1.1. Reduced Insulin Signaling and Brain Glucose Hypometabolism in AD and Other CNS Disorders

Although the brain makes up a very small percentage of the total body weight, it utilizes about 20% of the total oxygen and calories to meet its metabolic needs [7]. With glucose being the main source of energy in the brain, the constant uptake and metabolism of glucose is necessary to ensure normal functioning of the cells. The absorption of glucose into cells is dependent on the uptake and binding of insulin to receptors on the cell surface. Insulin signaling is responsible for effective glucose uptake and metabolism, without which the cell would have insufficient energy to function normally. Type 2 Diabetes Mellitus, hyperinsulinemia, and both systemic and brain insulin resistance, all of which show dysregulation of insulin signaling, increase the susceptibility to developing AD [8].

Brain glucose hypometabolism has been observed in a variety of CNS diseases (Table 1). It has been hypothesized that the lack of glucose uptake and metabolism in the brain would lead to a loss of cellular energy resulting in the inability of the brain to carry out its normal functions and to maintain homeostasis which could ultimately lead to cell degeneration [6].

### 1.2. Brain Iron Accumulation in AD and Other CNS Disorders

Iron is essential for brain function as it is necessary for processes such as oxygen transport, neurotransmission, and myelin homeostasis, amongst others [9]. Iron is mainly bound to iron-binding proteins in a cell. However, iron can also be found in its free state where it is not associated with proteins, and in this state, it is referred to as labile iron [10]. Labile iron catalyzes the Fenton reaction, which results in the formation of reactive oxygen species (ROS) that can cause oxidative stress and oxidative damage within the cell [11]. Excessive labile iron accumulates in specific regions of the brain in various brain diseases (Table 1) where it contributes to the neuroinflammation characteristic of many CNS disorders.

Ferroptosis is an iron-dependent cell death pathway observed in AD and other neurodegenerative diseases [12,13]. Ferroptosis is morphologically, biochemically, and genetically distinct from apoptosis, necrosis, and autophagy and requires iron-dependent accumulation of lipid ROS and leads to cell death [11,12]. Although ferroptosis continues to be explored in terms of its mechanism, chelating labile iron can help to prevent neuronal death via ferroptosis in AD.

## 2. BGHM and BIA Occur in Many CNS Disorders

BGHM and BIA occur in a number of CNS disorders. Table 1 presents a summary of many CNS disorders that show BGHM and BIA as demonstrated by fluorodeoxyglucose positron emission tomography (FDG-PET) and quantitative susceptibility mapping magnetic resonance imaging (QSM-MRI).

**Table 1:** CNS disorders with BGHM and/or BIA as demonstrated by FDG-PET and QSM-MRI, respectively. “✓” indicates that the respective biomarker has been reported in humans with a range of neurological disorders as compared to healthy controls.

CNS Disorder	BGHM	BIA
Addiction (alcohol and heroin)	✓ [14,15]	✓ [16,17]
Amyotrophic lateral sclerosis	✓ [18]	✓ [19]
Alzheimer’s disease	✓ [6]	✓ [6]
Corticobasal degeneration	✓ [6]	Insufficient literature [20]
Dementia with Lewy Bodies	✓ [21]	Insufficient literature [22]
Epilepsy	✓ [23]	✓ [24]
Fronto-temporal dementia	✓ [25]	✓ [26]
Ischemic stroke	✓ [27]	Insufficient literature
Multiple sclerosis	✓ [28]	✓ [29]
Multiple system atrophy	✓ [30,31]	✓ [32]
Parkinson’s disease	✓ [6,33]	✓ [6]
Post-traumatic stress disorder	Insufficient literature [34,35]	Rat (insufficient lit.) [36]
Progressive supranuclear palsy	✓ [37]	✓ [38,39]
Spinal cord injury	Neuropathic pain post-SCI [40,41]	Insufficient literature
Spinocerebellar ataxia 3	✓ [42]	✓ [43]
Traumatic brain injury	Insufficient literature [44,45]	✓ [46]
Vascular dementia	Insufficient literature	✓ [47]
Vascular parkinsonism	✓ [48]	Insufficient literature

From Table 1, it is evident that BGHM and BIA contribute to neurological dysfunction in a wide range of CNS disorders, and treatments targeting these biomarkers may benefit a large population of patients.

## 3. BGHM and BIA Are Also Involved in Multiple Aspects of AD Pathology

### 3.1. Decreased Acetylcholine Signaling and NMDA

The two classes of drugs that are currently prescribed to patients diagnosed with AD are cholinesterase inhibitors and n-methyl d-aspartate (NMDA) antagonists [49]. It is hypothesized that a decrease in acetylcholine (ACh) binding to receptors can contribute to the memory loss characteristic of AD. Acetylcholinesterase inhibitors are prescribed to reduce the degradation of ACh, and these are effective in slowing down the loss of cognitive function. Free iron catalyzes Fenton chemistry, which increases the generation of reactive oxygen species (ROS), causing oxidative stress. Both free iron and free heme, which are elevated in the brain in AD, have been shown to inactivate the human brain muscarinic cholinergic receptor in vitro [50]. Therefore, BIA can contribute to the breakdown of cholinergic signaling. The other class of drugs prescribed for AD are NMDA antagonists. The overactivation of NMDA receptors leads to excessive Ca^2+^ influx and, as a result, an overstimulation of glutamatergic neurons. Due to glutamate being excitatory, it results in excitotoxicity, synaptic dysfunction, and neuronal cell death. Insulin receptors have been identified on glutamatergic neurons. Insulin potentiates NMDA receptors in neurons and, as a result, plays a role in synaptic transmission and plasticity and in long-term potentiation.

### 3.2. Hyperphosphorylated Tau

Hyperphosphorylated tau (pTau) is a major research focus in the field of AD treatment. On accumulation, pTau forms neurofibrillary tangles, the progression of which has been shown to have a strong correlation with disease severity and memory loss [5]. BIA and pTau pathways are linked via glycogen synthase kinase 3β (GSK3β), an enzyme that phosphorylates tau. Deferoxamine, an iron chelator, has been demonstrated to decrease the activity of GSK3β, and the inhibition of GSK3β leads to a repression of tau hyperphosphorylation and blockage of tau deposition [9]. GSK3β is also downregulated in response to insulin [51].

### 3.3. Amyloid Beta

For many years, the prevailing amyloid hypothesis was that accumulation of amyloid beta (Aβ) and its subsequent formation of extracellular plaques leads to brain cell death in AD [2]. A 2015 paper summarized some of the main reasons for looking beyond the amyloid hypothesis in treating AD [52]. This paper mentions that the Aβ hypothesis is much better at explaining the heritable early-onset AD and extrapolating it to explain sporadic AD is likely incorrect. Therefore, this might be one of the primary reasons that treating sporadic AD (which makes up over 95% of AD cases) with treatments targeting Aβ does not lead to amelioration of AD symptoms.

The uptake and metabolism of glucose in many brain regions is dependent on the presence of insulin, and insulin signaling triggers the release of the insulin-degrading enzyme (IDE) in microglia and neurons. IDE is not only involved in degrading insulin but also in degrading Aβ, so reduced insulin signaling that can result in BGHM, can also result in reduced A-beta degradation. Further, both free iron and heme are elevated in the brains of people with AD [53,54]. Heme has been shown to form an A-beta-heme complex which has peroxidase activity [55].

### 3.4. Neuroinflammation

Inflammation is another of the major contributors to brain tissue atrophy observed in AD. The abnormal accumulation of iron in the CNS, which contributes to neuroinflammation, has been suggested to occur early in the course of neurological disorders [56]. The activation of brain immune cells such as microglia and astrocytes is the primary cause of inflammation, and changes in the activation profile of microglia and increased release of inflammatory cytokines are thought to induce insulin resistance [57]. As described previously, BIA can cause a disruption in redox homeostasis and lead to increased spread of ROS, ultimately leading to oxidative damage and neuroinflammation. Iron chelators such as deferoxamine are known to have anti-inflammatory effects. Additionally, intranasal insulin is anti-inflammatory [9]. Therefore, treating BGHM with insulin can also alleviate neuroinflammation, as insulin reduces inflammation by regulating the activation of the NLRP3 inflammasome [58].

## 4. Therapeutics Targeting BGHM or BIA

### 4.1. Intranasal Insulin for BGHM, Intranasal Deferoxamine (DFO) for BIA, and Other Intranasal Treatments for CNS Disorders

One of the major challenges with administering and targeting therapeutics to the brain has been the inability to deliver a wide range of active pharmaceutical ingredients and biologics to the brain due to the high selectivity of the blood–brain barrier. Some therapeutics can act not only on the brain but also on other organs, which could result in unwanted systemic side effects. However, the discovery of the intranasal (IN) method of administering therapeutics to the upper third of the nasal cavity to bypass the blood–brain barrier and target the brain via the olfactory and trigeminal neural pathways has demonstrated the possibility of delivering and targeting a wide variety of therapeutics to the brain to treat CNS disorders [34,59,60]. Here, we discuss the potential of repurposing two drugs approved for use in humans, insulin and deferoxamine, as therapeutics for CNS disorders.

Intranasal insulin (INI) has been demonstrated to safely improve memory in 20 min after administration in a clinical trial of human AD patients, and over a 21-day period, it improved memory in AD patients [61,62]. Intranasal insulin also improved memory in patients with Type 2 Diabetes, a disease that doubles the risk for AD [63]. The treatment has also been effective at decreasing the progression of white matter hyperintensities that are observed in higher volumes in AD, although no improvement in memory was observed in this clinical trial [64]. In multiple clinical trials conducted with cognitively normal adults, intranasal insulin also safely improved memory without altering blood levels of insulin or glucose [65,66].

INI acts on multiple pathways. Insulin promotes the uptake of glucose by brain cells by allowing transport of the GLUT 4 receptor to the cell surface. A 31P-MRI study showed that intranasal insulin administration increased energy (ATP and phosphocreatine) in brain cells in normal healthy humans [67]. Intranasal treatment of the intracerebroventricular-streptozotocin (ICV-STZ)-induced mouse model of Alzheimer’s disease with the insulin sensitizer metformin improved learning and memory without altering the blood levels of glucose [68]. As stated before, the presence of insulin increases the production of insulin-degrading enzyme, which plays a role in degrading amyloid beta, a biomarker that has extensively been used to characterize the disease.

A clinical trial demonstrated the safety and efficacy of INI in PD [69]. Intranasal insulin improved verbal fluency and movement while reducing physical disability when compared to the control group treated with placebo [69]. A 2021 study found that INI improves mitochondrial function and ameliorates motor deficits in the 6-OHDA rat model of PD [70]. It is clear that more research is needed to understand the benefit of INI in many of the mentioned CNS disorders, especially given that Table 1 shows that BGHM has been observed in these diseases. In summary, intranasal insulin safely bypasses the blood–brain barrier, increases brain cell energy, reduces inflammation, attenuates the HPA axis response to stress, improves memory, and reduces white matter hyperintensity in Alzheimer’s without altering the blood levels of insulin or glucose [58,64,67,71,72]. Intranasal insulin also causes improvements in T2D, Parkinson’s, and healthy adults in clinical trials, and is a potential treatment for TBI, SCI, PTSD, and certain other CNS disorders (see Table 2) [63,65,69].

Intranasal iron chelators also have significant therapeutic potential as they can bind free iron in the brain, effectively aiding in reducing oxidative stress and ferroptosis. One such iron chelator that has been tested in AD is deferoxamine (DFO). In 2012, it was demonstrated that APP/PS1 mice treated with IN DFO showed reduced spatial memory loss in the Morris water maze, and IN DFO was shown to reverse iron-induced memory loss in the same mouse model in 2013 [73,74]. Improved performance in radial arm water maze was also observed in P301L mice, a model for tauopathy [75]. In the streptozotocin rat model of AD, mice treated with IN DFO performed significantly better in Morris water maze and tapered balance beam test, demonstrating better spatial memory and fine motor control [76]. A 2020 study revealed that IN DFO showed improved memory in cognitively normal C57 mice [77]. Given the potential of IN DFO in treating mouse models of Alzheimer’s disease, future clinical trials will be beneficial in fully understanding its utility in treating AD. IN DFO has also been shown to be effective in treating rodent models of ischemic stroke and PD (Table 2). However, as seen from Table 1 and Table 2, many CNS disorders that have shown BIA are yet to be explored to understand if IN DFO administration can ameliorate the symptoms of the disease.

In humans, the safety and efficacy of DFO were first assessed in 1991, in a clinical trial of intramuscular desferrioxamine (deferoxamine) in individuals with probable AD. The study reported that the treatment resulted in a significantly lower rate of decline of daily living skills compared to the no-treatment group, and the no-treatment group showed a twice-as-rapid mean rate of decline [78]. The primary side effects reported by this study were loss of appetite and weight. In 1989, a small study in humans with iron overload secondary to chronic transfusion therapy reported that treatment with high doses of IN DFO in these individuals resulted in few side effects, mainly mild nasal irritation and an unpleasant taste in the mouth [79]. Carefully designed and controlled clinical trials will be needed to assess the safety and efficacy of intranasal deferoxamine in patients with CNS disorders.

**Table 2:** Evidence for the benefit of treating BGHM with INI or BIA with IN DFO in a range of neurological disorders tested in specific animal models. “Potentially” indicates that there is research hypothesizing that the treatment will be beneficial.

CNS Disorder	Evidence for Benefit of INI	Evidence for Benefit of IN DFO
Alzheimer’s disease	Human [61,62,65,80]	Mouse [76]
Epilepsy	Mouse [81]	No literature
Frontotemporal dementia	No literature	Mouse [75]
Ischemic stroke	Potentially [82]	Rat [83]
Parkinson’s disease	Human [69]	Rat [84]
Post-traumatic stress disorder	Potentially [85]	Potentially [36]
Spinal cord injury	Potentially (rat) [86,87]	Rat [88]
Traumatic brain injury	Rat [89]	No literature
Vascular dementia	Rat [90]	Potentially [91]

Thus far, no one has published results for the effects of INI or IN DFO in the following disorders in humans or animal models: addiction, dementia with Lewy bodies, vascular parkinsonism, progressive supranuclear palsy, corticobasal degeneration, amyotrophic lateral sclerosis, spinocerebellar ataxia 3 and multiple sclerosis. However, as shown in Table 1, many of these disorders exhibit BGHM and/or BIA providing a rationale for testing the safety and efficacy of INI and/or IN DFO.

### 4.2. Insulin-Modulating Drugs: Metformin, Thiazolidinediones, Sodium-Glucose Cotransporter-2 Inhibitors, Glucagon-Like Peptide-1

Recent studies have demonstrated many insulin-modulating drugs to have efficacy in treating AD [92].

Metformin, one of the primary insulin-sensitizing medications prescribed to patients with Type 2 Diabetes Mellitus (T2DM), improved memory in patients with AD and no history of diabetes or pre-diabetes [93]. Pioglitazone, a member of the class of medications known as thiazolidinediones, has also been shown to improve dementia in patients with AD [94]. A 2020 paper found that sodium-glucose cotransporter-2 (SGLT-2) antagonist Empagliflozin reduced vascular damage and cognitive impairment in AD-T2D mice showing potential for treatment [95]. Glucagon-like peptide-1 (GLP-1) receptor agonists are a family of drugs that are used in the treatment of T2DM, and use of these has shown benefit in treating pathologies of AD and PD in phase II clinical trials [96]. A recent study targeted both BGHM and BIA in a mouse model of PD using a treatment that contained a GLP-1 receptor agonist, Exendin-4, and the iron chelator DFO [97]. The study reported that intranasally administered nanoparticles loaded with Exendin-4 peptide-conjugated DFO, synergistically ameliorated dopamine neuron loss and neuroinflammation in the substantia nigra and motor deficits in the mice. Future studies testing the effectiveness of these treatments in diseases other than AD and PD can help to determine the CNS disorders that may benefit from these treatments.

## 5. Limitations and Future Directions

Clinical trials of INI to date have not revealed major adverse side effects (Section 4.1). However, because brain glucose hypermetabolism has been observed in certain brain regions of some neurological disorders, it will be important to determine if the presence of hypermetabolism in those brain regions is a contraindication to the treatment of BGHM in other brain regions [18]. Insulins designed for use in treating diabetes contain preservatives, which may be responsible for the mild nasal irritation reported by some participants in clinical trials. Elimination of the need for preservatives by creating unit dose nasal sprays or by other means could help to alleviate this problem.

There is a need for clinical trials assessing the safety and efficacy of therapeutics targeting BGHM and BIA in the neurological disorders discussed in Table 1. In addition, assessing the efficacy of agents targeting BGHM in combination with agents targeting BIA for additive or synergistic effects is essential.

## 6. Conclusions

BGHM and BIA are promising therapeutic targets beyond their utility in monitoring disease progression or in contributing to distinguishing various CNS disorders based on brain biodistribution of the biomarkers. BGHM and BIA are observed across a large range of CNS disorders, and treatments targeting them have demonstrated therapeutic potential in preclinical studies and for intranasal insulin in clinical trials for AD and PD. With more experimental evidence and clinical trials demonstrating safety and efficacy, these treatments can also be repurposed to treat other CNS disorders that show BGHM and/or BIA.

## Abbreviations

The following abbreviations are used in this manuscript:

BGHM	Brain glucose hypometabolism
BIA	Brain iron accumulation
AD	Alzheimer’s disease
CNS	Central Nervous System
ROS	Reactive oxygen species
FDG-PET	Fluorodeoxyglucose positron emission tomography
QSM-MRI	Quantitative susceptibility mapping magnetic resonance imaging
NMDA	N-methyl d-aspartate
ACh	Acetylcholine
pTau	Hyperphosphorylated tau
GSK3β	Glycogen synthase kinase 3β
Aβ	Amyloid beta
IDE	Insulin-degrading enzyme
IN	Intranasal
INI	Intranasal insulin
DFO	Deferoxamine
TBI	Traumatic brain injury
SCI	Spinal cord injury
PTSD	Post-traumatic stress disorder
GLP-1	Glucagon-Like Peptide-1
T2DM	Type 2 Diabetes Mellitus
SGLT-2	Sodium-glucose cotransporter-2
PD	Parkinson’s disease
